# High false hepatitis C antibody positivity rate in a regionally-inclusive population of non-renumerated blood donors in Uganda

**DOI:** 10.4314/ahs.v24i3.6

**Published:** 2024-09

**Authors:** P Ocama, R Ssekitoleko, J Nankya-Mutyoba, B Apica, G Otekat, E Seremba

**Affiliations:** 1 School of Medicine, Makerere University College of Health Sciences, Kampala, Uganda; 2 School of Public Health, Makerere University College of Health Sciences, Kampala, Uganda; 3 Uganda Blood Transfusion Service, Kampala, Uganda

**Keywords:** High false hepatitis C, antibody positivity rate, non-renumerated blood donors, Uganda

## Abstract

**Background:**

Successful elimination of hepatitis as a public health threat by 2030 will partly rely on the availability and accessibility of affordable accurate disease testing platforms. In the past, testing of hepatitis C virus (HCV) in low resource settings of sub-Saharan Africa (SSA) has relied on anti-HCV testing using rapid diagnostic tests, chemiluminescent microparticle immunoassay (CMIA) and Enzyme-linked Immunosorbent Assays (ELISA) whose diagnostic accuracy has been sub-optimal. We determined the false positivity rate of a CMIA platform that is routinely used to screen donor blood for anti-HCV in Uganda.

**Methods:**

1,216 CMIA-screened anti-HCV-positive blood donor samples at four regional Ugandan blood banks, were subjected to a third generation ELISA and subsequently to nucleic acid testing (NAT).

**Results:**

Of the above 1,216 samples, 1,122 (92.2%) were negative on ELISA and thus deemed false positives. Active infection (NAT positive) was detected in 98 (8.0%). Presumed resolved infection was recorded among 3 (3.2%) of participants that remained positive on the ELISA platform but negative on NAT.

**Conclusion:**

The Architect CMIA assay exhibited very low specificity for anti-HCV testing. In this context, this finding may suggest need to employ testing protocols that include NAT or a combination of tests with higher validity.

## Introduction

Hepatitis C virus (HCV) infection is a recognized public health challenge affecting an estimated 58 million individuals worldwide^1^. Recent World Health Organization (WHO) estimates suggest that over 1% (≥9.1 million) of the African population is infected with this disease^2^, superseded by European 1.3% (12million), East Mediterranean 2.3% (12million), South-East Asia 2.3% (10million) and the Western Pacific regions 0.5% (10million) populations. HCV infection is associated with a high mortality. In 2019, approximately 290,000 deaths occurred worldwide from HCV-related complications of cirrhosis, liver cancer and liver failure^1^. In Africa, the Center for Disease Analysis Foundation estimates that this disease claims a life every 5.5 minutes^3^ and its incidence is higher in Africa than in America, Southeast Asia and in the western Pacific region^4^. Triggered by the high mortality and morbidity associated with viral hepatitis, the WHO launched a campaign to eliminate these diseases by the year 2030. This campaign seems to be rather slow in Africa where some implementation of interventions including full vaccination against hepatitis B virus (HBV), prevention of mother-to-child transmission of HBV and injection safety, harm reduction as well as testing and treating HBV/HCV that are considered to be of high impact in eliminating these diseases have not been fully implemented as yet^5^.

Fortunately, HCV is curable and could be prevented. Harm reduction among persons who inject drugs, use of non-reusable needles and screening of donor blood play significant roles in preventing transmission. However, testing and treating HCV require accurate identification of infected persons. The majority of sub-Saharan African countries are yet to conduct mass population screening campaigns for HCV partly due to lack of resources. Routine clinical care and in some settings, screening of donor blood for HCV in Africa is performed using Rapid Diagnostic Tests (RDTs), chemiluminescent microparticle immunoassay (CMIA) and Enzyme-linked Immunosorbent Assay (ELISA) platforms. RDTs have been traditionally preferred in settings with limited laboratory services as they are relatively inexpensive, easy to use and facilitate linkage to care. Their performance and that of ELISA has however previously been found to vary and sometimes is sub-optimal in certain African settings including blood banks. In previous studies of hospitalized Uganda adults, the RDTs demonstrated poor diagnostic accuracy for HCV on the emergency medical ward^6^ and a high false positivity rate in a hospitalized sub-population of HIV-infected individuals^7^. In the blood bank settings in Kaduna, Nigeria, the pre-donation screening tests of donor blood demonstrated low sensitivity for HCV infection in comparison to the ELISA platform^8^. In high income countries, antibody tests are supplemented with Nucleic Acid Tests (NAT) to confirm active HCV infection. More recently, the WHO proposed Point-of-care (POC) HCV RNA viral load assays as an alternative approach to laboratory-based HCV RNA NAT assays for the diagnosis of HCV viraemic infection^9^. It however remains unclear how soon its implementation will be effected in the low resource and hard to reach settings of Africa.

Currently, in the Uganda Blood Transfusion Service (UBTS), antibody testing is neither supplemented by nucleic acid nor hepatitis C core antigen testing. More recently, a validation study of anti-HCV chemiluminescent microparticle immunoassay (CMIA) on the Architect i2000SR analyzer (Abbott Diagnostics, Germany), a FDA/WHO approved assay that is routinely used to screen donor blood for anti-HCV in Uganda against the SD Biosensor and HCV cAg platforms in an algorithmic approach demonstrated a very high false positivity rate^10^. This inevitably raises anxiety among blood donors and leads to wastage of potentially safe blood for transfusion but could also pose a risk of transmitting HCV to the recipients^8^. In a regionally-inclusive sample of blood donors in Uganda, our study aimed to assess the false positivity rate of the above national blood bank HCV antibody (anti-HCV) screening assay against ELISA and NAT platforms and to characterize the HCV prevalence among the blood donors using NAT as a reference standard.

## Methods

### Study design, setting and data collection

This study utilized a cross-sectional design. It was conducted at the UTBS, a semi-autonomous, centrally coordinated organization in the Ministry of Health of Uganda that is mandated to provide sufficient and efficacious blood and blood components for appropriate use in health care service delivery in Uganda. It recruits volunteer non-renumerated blood donors across its regional branches in West Nile, Western, Southwestern, Northern, Eastern and Central regions of Uganda. Donor blood is screened for transfusion transmissible infections including HIV, syphilis, hepatitis B and hepatitis C using a multiple test system Architect ci8200-system (Abbott, Weisbaden, Germany). In line with blood bank protocol, hepatitis C (anti-HCV) positive samples are taken through second line testing using Murex anti-HCV ELISA, V 4, (Diasorin, South Africa). If this is still positive, the sample is declared positive and blood is discarded.

For this study, we included blood donors aged ≥18 years, who have tested positive for anti-HCV on the CMIA assay and who consented to participate in the study.

### Laboratory procedures and analyses

Potential participants were called back to the regional blood banks by the bank counselors to receive their results. At the same time, they were informed about the study and those who accepted and consented to take part in the study had 20 mls of blood taken in a purple top container for further analysis. The samples were transferred on the same day to a regional branch of an internationally certified clinical diagnostic laboratory (MBN clinical laboratory). At the MBN laboratory, these samples were processed and stored at a temperature of 20 degrees Celsius. They were assayed with another laboratory-based ELISA for anti-HCV antibodies using the AccuDiag™ HCV ELISA Assay Catalog No. 1707-12 (Diagnostic Automation/Cortez Diagnostics Inc., California, USA) platform which according to the manufacturer has a sensitivity and specificity >98% for anti-HCV. Subsequently, qualitative and quantitative nucleic acid testing for HCV were conducted.

Qualitative RNA PCR was conducted on all samples using a real time PCR assay - the AmpliSens HCV-FRT assay Catalog No. R-V1-Mod (RG,iQ,Mx,Dt)-CE (Ecoli s.r.o. Bratislava, Slovenská republika). PCR assay was performed through RNA extraction from the sample with exogenous internal control, RNA reverse transcription and simultaneous amplification of cDNA fragments with real time hybridization-fluorescence PCR detection. All procedures were conducted following the manufacturer's instructions. On the other hand, quantitative HCV viral load testing was performed on qualitative-HCV PCR-positive samples using Roche COBAS 6800HCV Test (Roche Diagnostics, Switzerland) whose lower limit for detection is 10 IU/mL.

Using the MBN testing platforms, anti-HCV ELISA test results were interpreted as ‘negative’ if the signal to-cut-off (S/CO) ratios was less than 1.0, and ‘positive’ if greater than or equal to 1.0. Similarly results of the qualitative RNA real time PCR test were interpreted as ‘negative’ if HCV RNA was not detected at all i.e. below the lower detection limit/Analytical sensitivity of 50 IU/ml or ‘positive’ if HCV RNA was detected.

Statistical analyses: Data were analysed to compute the false positivity rate and positive predictive value of the CMIA-based HCV-screening blood bank protocol using the MBN lab ELISA (AccuDiag HCV ELISA) platform as the gold standard. False positivity was defined by a positive anti-HCV CMIA screened sample; negative on ELISA. Active infection was defined as a positive NAT test and was measured as a proportion of NAT positive samples among the total number of anti-HCV positive samples as determined by the above blood bank CMIA and the reference lab ELISA protocol respectively. Presumed resolved infections were those that tested anti-HCV positive on the ELISA platform and negative on NAT testing.

Ethical clearance: This study received ethical review and clearance from Makerere University College of Health Sciences School of Medicine Research and Ethics committee (SOMREC, REF REC 2018 184) and the Uganda National Council of Science and Technology (UNCST, SS 4870). The study was conducted in strict conformity with the declaration of Helsinki and all participants provided consent for participation and for sample storage. Participant confidentiality was kept during consenting and data collection. Participants who were found to be HCV-infected were referred to hepatitis clinics at the nearest regional referral hospitals for treatment.

## Results

Of the 1,216 samples that were positive for anti-HCV using the blood bank CMIA, 1122 (92.2%) turned out negative on the reference ELISA platform and were deemed to be false positives, resulting in a CMIA positive predictive value of 0.08. Active HCV infection (HCV viral load) was recorded in only 8.0% (98/1216) of the participants, seven of whom had tested negative for anti-HCV on the ELISA. In addition, basing on ELISA as a Gold standard for anti-HCV testing, and NAT testing, resolved HCV infection was detected among 3.2% (3/94) of the samples in whom antibodies were detected but had no detectable HCV RNA. All the samples that were deemed positive using the qualitative NAT testing were confirmed positive by the quantitative NAT assay.

## Discussion

This study has unveiled a high false positivity rate of the WHO/FDA approved Architect chemiluminescent microparticle immunoassay (CMIA) platform for HCV antibody diagnosis^11^ among non-enumerated blood donors in Uganda. This finding is similar to earlier observations among hospitalized HIV-seronegative and seropositive adults in Uganda and other sub-Saharan African countries where other anti-HCV testing platforms, Diagnostic Tests (RDTs) and ELISA platforms had both a poor sensitivity and specificity for anti-HCV detection 12-16. Relatedly, a recent study in the same blood bank using a combination of the bank protocol and the WHO-recommended HCV cAg as alternative to NAT also found a low sensitivity of the above Architect assay^10^. The current (Architect) and previous (ELISA/RDTs) assays validated for HCV testing in Uganda therefore demonstrate performance characteristics that fall short of the minimum recommended sensitivity and specificity of >98% and >97% for RDTs and 100% and >98% for ELISAs^17^. Our reference lab ELISA exhibited a much better validity for anti-HCV testing. ELISA platforms have however demonstrated good performance in European and American populations^18^, and in a study among HIV-infected adults in Gabon, Central Africa^19^, suggesting need for validation of the third but preferably the fourth generation ELISA assays in populations across geographical locations before use in blood bank and clinical settings. Alternatively, the use of a combination of assays could potentially improve pathogen detection among blood donors and high-risk populations for HCV infection 19, 20. This is of utmost importance in SSA where there is a rapidly growing population, high HIV prevalence, meagre resources for confirmatory testing of anti-HCV positive individuals, overwhelmed human resource to handle the unnecessary anti-HCV positive referrals, a critical shortage of safe donor blood 21, 22 and slow response to the WHO-led campaign to eliminate HCV by 2030^11^. As more efforts are being directed towards the production of more accurate diagnostics, evaluating the performance of the newer fourth-generation ELISA HCV assays in African populations and the use of HCVRNA point of care tests could potentially provide a solution to some of these challenges 9. Contrary to studies in other settings where resolved infections constitute ≥20% of all antibody-positive samples^23^,^24^, only 3.2% of our participants had resolved HCV infection. In Uganda and some other African settings findings suggest that false positive HCV antibody test results are common and could be attributed to cross reacting antigens^6^, ^25^-^27^.

This study has some limitations. Only blood samples that were anti-HCV positive were included in this study. Those that were indeterminate were not available for further evaluation. Despite this limitation, we evaluated a large number of samples that were collected across the geographical regions of the country in a way that is similar to that employed in routine clinical care. They are therefore representative of what the national HCV screening picture would be like. In addition, we conducted nucleic acid testing to confirm the presence of infection.

## Conclusion

We found a very high false anti-HCV positivity rate with the Architect CMIA platform. In the context of blood donor testing this could suggest a need for investment in better diagnostic platforms or at least a combination of assays in an algorithmic approach in order to better understand the disease epidemiology and resources needed for its screening and treatment if the WHO-led goal of eliminating HCV by 2030 is to be met.

## Figures and Tables

**Figure 1:**
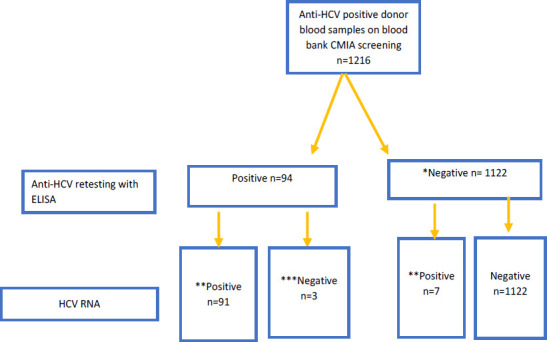
Hepatitis C virus results following repeat (ELISA anti-HCV) and subsequent (HCV nucleic acid) testing *Presumed false positives: Positive on blood bank CMIA, negative on ELISA testing n=1122 (92.2%) **Active infection: Positive for HCV RNA on NAT testing n=98 (8.0%) regardless of the anti-HCV test result. Positive on both CMIA and NAT testing n=98 (8.0%), ELISA and NAT n=91 (7.4%), negative on ELISA but positive on NAT testing n=7/1122 (0.62%). ***Resolved infection: Positive on reference lab ELISA, negative on NAT testing n=3/94 (3.2%)
